# Immediate postplacental insertion of a copper intrauterine device: a pilot study to evaluate expulsion rate by mode of delivery

**DOI:** 10.1186/s12884-015-0637-6

**Published:** 2015-09-02

**Authors:** Ayhan Sucak, Sarp Ozcan, Şevki Çelen, Turhan Çağlar, Gonca Göksu, Nuri Danışman

**Affiliations:** Department of Perinatology, Dr Zekai Tahir Burak Research and Training Hospital, Ankara, Turkey; Department of Obstetrics and Gynecology, Acıbadem Hospital, Turan Güneş Bulvarı 630.sokak No:6 Or-an, Ankara, Turkey

## Abstract

**Background:**

The present study aimed to investigate risk factors for expulsion in immediate postplacental IUD insertion. We specifically sought to determine whether cesarean delivery before or during labor have an impact on IUD expulsion.

**Methods:**

The study included 160 pregnant women for immediate IUD insertion following vaginal or cesarean delivery. Three groups of patients were recruited: Patients who underwent pre-planned cesarean delivery (group 1, n: 51), patients who underwent cesarean delivery during active labor (group 2, n: 47), patients who delivered vaginally (group 3, n: 62).

**Results:**

The cumulative expulsion rates were similar with a frequency of 8.7, 8.9 and 11.3 % respectively in groups 1 to 3 (*p* > 0.05 in all pairwise comparisons). The rate of patients who had the IUD removed at 12th month was 4,3, 6.7 and 11.3 % for groups 1, 2 and 3 respectively (*p* > 0.05 in all pairwise comparisons). Multiparity increased the risk of cumulative expulsion within 12 months by 2.1 fold (95 % 1,03–4,37) in the logistic regression model. Previous vaginal deliveries or IUD use did not have an impact on the expulsion of the IUD. The risk of spontaneous expulsion was similar in patients whose IUD was placed after cesarean in the active and latent phase or after spontaneous vaginal delivery.

**Conclusions:**

The rates of IUD expulsion are similar in patients who underwent cesarean section before and during labor and who delivered vaginally. Parity was the only factor independently associated with IUD expulsion.

## Background

An intrauterine device (IUD) is a coitus-independent, reversible and effective form of contraception with immediate contraceptive action. It is the most widely used method of contraception with approximately 160 million users worldwide [1–3]. Globally, 14.3 % of female contraceptive users prefer the IUD [4]. Previous data indicate that IUDs are as effective as tubal sterilization [5]. Moreover, despite the well-known complications such as increased menstrual bleeding and pain long term discontinuation rates are generally low [1, 2, 6].

Immediate postplacental IUD insertion is defined as placement of an IUD within 10 min following delivery. Insertion during this period is associated with less discomfort; and puerperal women may have increased motivation for contraception [7]. Most studies have found immediate postplacental IUD insertion to be safe and effective [6–13]. Cumulative expulsion rates 12 months after vaginal delivery have been reported to be between 13 and 19 % [8, 9]. Expulsion rates 12 months after caesarean delivery are generally lower and have been reported to be between 9 and 14 % [8, 9]. According to some studies, the risk factors for IUD expulsion following immediate postplacental insertion were vaginal delivery and parity [8, 9, 12].

Although immediate postplacental IUD insertion is a viable contraceptive option, there are relatively few data about the risk factors for IUD expulsion. A higher expulsion rate following vaginal delivery may be anticipated as a result of cervical dilation as well as development of the thin lower segment during labour. No study, however, has investigated the impact of labour on the risk of expulsion in patients with immediate postplacental IUD insertion.

In the present study we aimed to investigate whether there was a difference in cumulative expulsion rates 12 months after immediate postplacental IUD insertion between patients who delivered vaginally and those who underwent caesarean section. We also investigated the risk factors for expulsion and specifically sought to determine whether caesarean delivery before or during labour had an impact on IUD expulsion.

## Methods

**Fig. 1:**
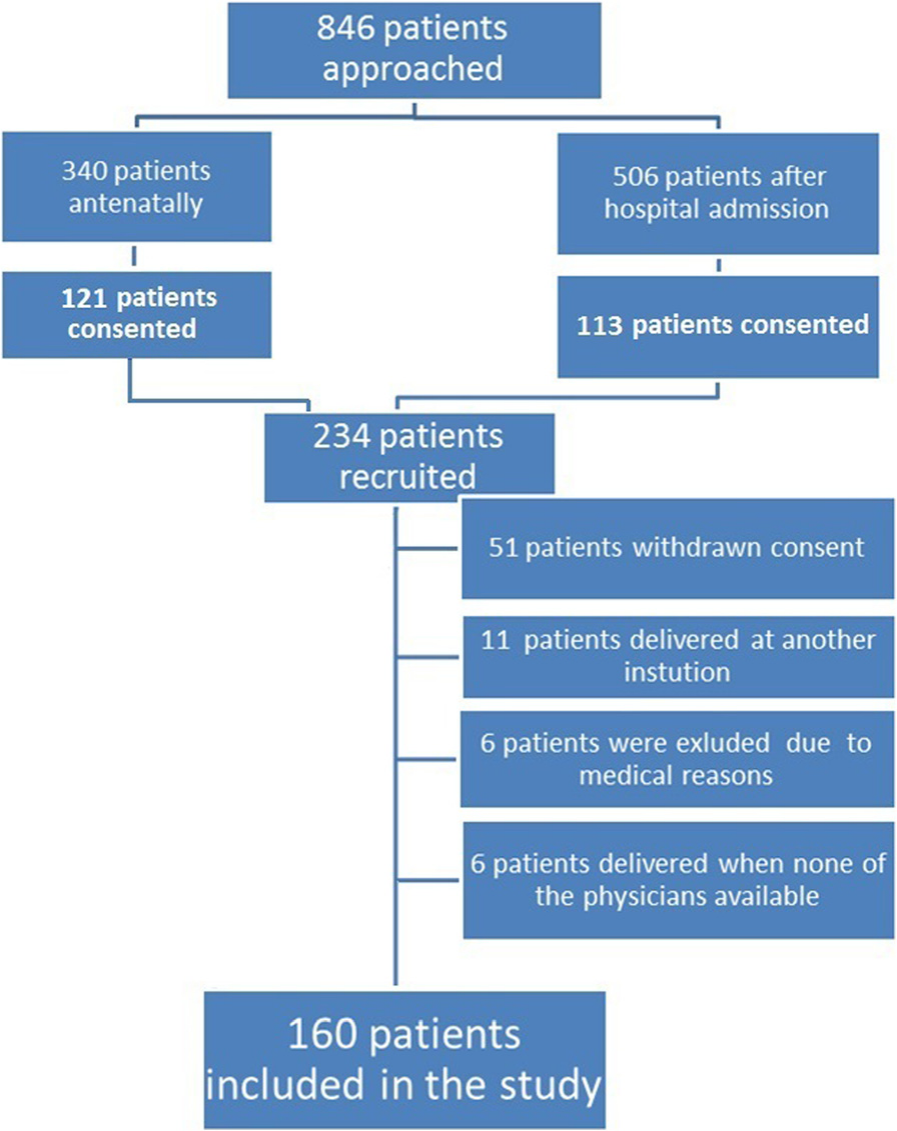
Recruitment of the study population

This prospective cohort study was conducted at Zekai Tahir Burak Research and Training Hospital, Ankara, Turkey, between January 2009 and June 2012. The study included pregnant women who were scheduled for immediate IUD insertion following removal of the placenta. All patients who chose the IUD as their contraceptive method and were willing to take part in a 1-year follow-up were invited to participate in the study. Three groups of patients were recruited: group 1 comprised patients who underwent preplanned caesarean section; group 2 comprised patients who underwent caesarean section during active labour due to obstetric indications; and group 3 comprised patients who delivered vaginally (Fig. 1). Active labour was defined as regular and painful uterine contractions accompanied by cervical effacement and dilatation of at least 1centimeters. Patients with multiple pregnancies, placenta previa, intrapartum fever, rupture of membranes for longer than 24 h, postpartum hemorrhage, or active untreated lower genital tract infection were not included in the study. In addition, patients with a history of ectopic pregnancy or those who required manual removal of the placenta were also not included.

An IUD (Pregna Copper T 380A; Pregna International, Chakan, India) was placed in the fundus of the uterus by one of the authors within 10 min of placental delivery. In patients who delivered by caesarean section, the IUD was placed through the hysterotomy incision using a ring forceps, and the strings were passed through the cervix; at 6 weeks postpartum, the strings were trimmed to extend just beyond the external cervical os. During caesarean section, all women received prophylactic intravenous cefazolin (2 g). In women delivering vaginally, the IUD was placed using the prepackaged inserter provided by the manufacturer. Using a sterile technique, the arms of the device were folded and the IUD was loaded at the distal end of the inserter. The plunger was loaded at the opposite end of the inserter and the IUD was than inserted under abdominal ultrasound guidance. Prior to discharge, all patients were re-examined, including abdominal ultrasonography. Patients were scheduled for a control examination at 6 weeks, 6 and 12 months postpartum and were instructed to contact one of the physicians (study authors) immediately if they experienced pelvic pain, fever, excessive bleeding or an unusual vaginal discharge. At each visit the patients were questioned for symptoms of expulsion and infection. In addition, a pelvic examination and transvaginal ultrasound were performed by one of the authors at each follow-up visit. Complete IUD expulsion was verified clinically and by transvaginal ultrasound. An IUD was considered to be partially expelled if a distance greater than 10 mm was measured between the vertical arm of the IUD and the junction between the endometrium and the uterine cavity 6 weeks postpartum. The IUD was removed in the case of partial expulsion, bleeding or pain, or at the patient’s request. Both complete and partial expulsions and are collectively referred as expulsions.

Sample size calculation was performed using G*Power software (Franz Faul, Kiel, Germany). The following assumptions were made for calculating the two-sided confidence interval (1-α): % 95, power (1-β): % 80. Based on previous data, the cumulative IUD expulsion rates after 12 months were assumed to be 19 % for patients with vaginal deliveries and 14 % for patients with caesarean deliveries [9]. With an allocation ratio of 2:1 (group 1 + group 2/group 3), a sample size of approximately 530 individuals per group was estimated to be necessary, assuming a 5 % difference in cumulative expulsion rates at 12 months. It was not, however, feasible to recruit such a large sample size, for the following main reason. During the recruitment period there were 44,959 deliveries. Assuming 5 % take-up of the IUD in our patients, one could expect 2250 potential IUD users. To achieve recruitment of 530 patients in each group more than 70 % of potential IUD users would have to be identified, which is virtually impossible without implementing a hospital-wide policy and unachievable due to the heavy workload at our hospital. We therefore intended to carry out a pilot study of 160 patients (approximately 50 patients in each group, assuming a drop-out rate of 5 %).

The following clinical data were collected from the patients: age at delivery, gravidity, parity, gestational age at delivery, mode of delivery, and indications for caesarean delivery in previous and current pregnancies. Patients were also questioned about their previous IUD use and desire for future fertility.

The study was approved by the ethics and educational issues coordinating committee of Zekai Tahir Burak Research and Training Hospital (2008–122). All patients gave their written consent to participate in the study and for their data to be used with appropriate ethics committee approval. Statistical analyses were performed using SPSS software, version 17 (SPSS, Chicago, IL, USA). One-way analysis of variance was performed for parametric variables between groups with a normal distribution. Pearson’s *χ*2 test and Fisher’s exact test were performed for nominal or ordinal variables between groups where appropriate. Multivariate stepwise logistic regression with backward elimination was performed to investigate certain parameters on cumulative risk of IUD expulsion within groups. In the logistic regression model, age and parity were included as continuous variables whereas presence of previous vaginal delivery and IUD use as categorical variables. A p-value less than 0.05 was considered significant.

## Results

We enrolled 160 patients into the study during the recruitment period. Patients in group 1 were recruited over 14 months, whereas patients in groups 2 and 3 were recruited over 30 months. During the recruitment period there were 44,959 deliveries, of which 21,195 (47.1 %) were caesarean. Parity and maternal and gestational age at delivery were similar between the groups. Previous IUD use and desire for future fertility were similar between the groups. The number of patients who had at least one previous vaginal delivery was higher in group 3 than in groups 1 and 2 (*p* < 0.01 for both pair wise comparisons; Table 1).

**Table 1:** Clinical characteristics of the patients with immediate postplacental IUD insertion

	Group 1 (cesarean delivery before labor)	Group2 (cesarean delivery during active labor)	Group3 (vaginal delivery)	p^1vs2^	p^1vs3^	p^2vs3^
	(*n* = 51)	(*n* = 47)	(*n* = 62)			
Maternal age (years)	27.7 ± 5.1	25.3 ± 5.2	26.6 ± 4.4		0.06	
Gestational age at delivery (weeks)	38.0 ± 1.2	38.8 ± 1.6	38.4 ± 2.4		0.14	
Parity						
0	11 (21.6 %)	11 (23.4 %)	11 (17.7 %)	0.78	0.79	0.62
1–3	40 (78.4 %)	34 (72.3 %)	48 (74.4 %)	0.24	0.90	0.70
≥4	0	2 (4.3 %)	3 (4.8 %)	0.23	0.25	1.0
Number of previous vaginal deliveries						
0	22 (43.1 %)	23 (48.9 %)	8 (12.1 %)	0.71	<0.01*	<0.01*
1–2	29 (56.9 %)	21 (44.7 %)	42 (67.7 %)	0.32	0.32	0.03*
≥3	0	3 (6.4 %)	12 (19.4 %)	0.11	<0.01*	0.25
Previous IUD use	29 (57 %)	22 (48 %)	40 (65 %)	0.43	0.53	0.08
Future fertility desire	29 (57 %)	30 (64 %)	31 (50 %)	0.53	0.59	0.21
Cesarean indications						
Non-progressive labor	0	26 (55 %)		<0.01*		
Fetal distress	4 (8 %)	18 (38 %)		<0.01*		
Recurrent cesarean delivery	37 (73 %)	0		<0.01*		
Malpresentation	5 (10 %)	3 (6 %)		0.80		
Fetal macrosomia	5 (10 %)	0		0.09		

Data expressed as mean ± SD. median (minimum - maximum) and number (%). IUD: Intrauterine device, * indicates *p* < 0.05

**Fig. 2:**
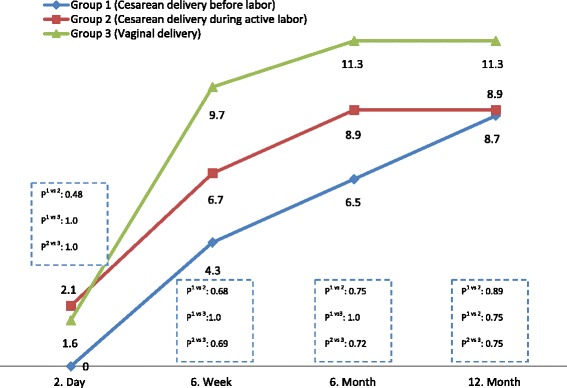
Cumulative rates of IUD expulsion in groups (%)

Spontaneous IUD expulsion rates are shown in Fig. 2. The cumulative expulsion rates had a similar frequency of 8.7, 8.9 and 11.3 % in groups 1, 2 and 3, respectively (*p* > 0.05 in all pair wise comparisons; Table 2). The expulsion rates at 6 weeks postpartum were 4.3, 6.7 and 9.7 % in groups 1, 2 and 3, respectively (*p* > 0.05 in all pair wise comparisons; Fig. 2). In each group, more than half of the expulsions occurred within 6 weeks postpartum.

**Table 2:** Cumulative rates of expulsion, discontinuation and patients lost to follow up in the study population

	Group 1 (cesarean delivery before labor)	Group2 (cesarean delivery during active labor)	Group3 (vaginal delivery)	p^1vs2^	p^1vs3^	p^2vs3^
	(*n* = 51)	(*n* = 47)	(*n* = 62)			
Rates of IUD removal at 12 months^a^	2 (4.3 %)	3 (6.7 %)	7 (11.3 %)	0.67	0.28	0.59
Bleeding	1 (2.2 %)	0	3 (4.8 %)	1.0	0.63	0.35
Pain	1 (2.2 %)	2 (4.5 %)	1 (1.6 %)	0.61	1.0	0.58
Planned pregnancy	0	1 (2.3 %)	1 (1.6 %)	0.48	1.0	1.0
Personal reasons	0	0	2 (3.2 %)	1.0	0.50	1.0
Expulsion rates at 12 months^a^	4 (8.7 %)	4 (8.9 %)	7 (11.3 %)	1.0	0.91	0.94
Complete expulsion	2 (4.3 %)	3 (6.7 %)	3 (4.8 %)	0.68	1.0	0.69
Partial expulsion	2 (4.3 %)	1 (2.2 %)	4 (6.5 %)	1.0	1.0	0.40
Lost to follow up	5 (9.8 %)	2 (4.3 %)	0	0.44	0.02*	0.18
Continuation rate at 12 months^a^	40 (87 %)	38 (84 %)	48 (77 %)	0.97	0.31	0.51

Data expressed as number (%), ^a^Patients who were lost to follow up are excluded from analysis. * indicates *p* < 0.05

There were no pregnancies in the study population while the IUD was in place. No significant side effects (e.g. infection or perforation) occurred during IUD placement. Seven patients were lost to follow-up during the study (five in group 1 and two in group 2). The cumulative rate of IUD removal in the whole study population was 7.8 %. The rates of IUD removal were similar in all groups (*p* > 0.05 in all pair wise comparisons; Table 2).

The risk factors for IUD expulsion are shown in Table 3. Multiparous patients had a 2.1-fold increased cumulative risk of expulsion (95 % CI 1.03–4.37) within a year after insertion. Previous vaginal delivery or IUD use did not affect IUD expulsion. The risk of spontaneous expulsion was also similar in women whose IUD was placed after caesarean section during active labour or after spontaneous vaginal delivery.

**Table 3:** Risk factors associated with spontaneous IUD expulsions following post-placental placement

Characteristics	Crude odds ratio	Confidence interval	Adjusted odds ratio	Confidence interval
Multiparity	1.27*	1.07–1.50	2.12*	1.03–4.37
IUD after cesarean during active labor	1.03	0.49–2.13	2.82	0.39–20.69
IUD after vaginal delivery	1.17	0.72–1.90	1.43	0.17–12.32
Previous vaginal delivery	1.40*	1.1–1.78	2.23	0.30–16.56
Previous IUD use	0.43	0.18–0.995	0.52	0.07–3.77

*IUD* Intrauterine device, * indicates *p* < 0,05

## Discussion

### Findings and interpretation

The findings suggest that the route of delivery (vaginal or caesarean) and the timing of caesarean section had no impact on spontaneous IUD expulsion. The cumulative expulsion rate, which was around 10 % within 12 months, was similar in all groups of patients. In addition, for the first time in the literature, we have demonstrated that active labour did not increase the risk of spontaneous expulsion in patients who had undergone caesarean section.

There were no unwanted pregnancies or an acute complication related to the insertion of the IUD in the current study suggesting that immediate postplacental insertion of Copper IUD is a safe and effective method which is in accordance with the previous reports [7, 8, 10]. The cumulative expulsion rate, which was around 10 % within 12 months in the current study is compatible with the previous reports, as the overall expulsion rate was calculated to be 9.5 % in a previous pooled analysis [14]. Furthermore, similar to our results, there are reports where a significant proportion of the expulsions occurred within the first 3 months after the insertion of IUD [7, 11]. We believe this is mainly due to the puerperal uterine remodeling.

### Differences in results and conclusions in relation to other studies

Only a few studies have addressed the mode of delivery in relation to subsequent IUD expulsion. A multicenter study, with the largest sample size so far, reported that expulsion rates were higher in patients who received an IUD after vaginal delivery [11]. The expulsion rates at 3 months were 10.9 % for IUDs placed after caesarean delivery and 16.4 % for IUDs placed after vaginal delivery; rates which are slightly higher than ours. A more recent study reported that the expulsion rate for IUDs placed immediately after vaginal delivery was 38 %, while only 12 % of IUDs placed after caesarean delivery were expelled [15]. Similar cumulative expulsion rates for postplacental insertion after caesarean and vaginal delivery have been reported [8, 9]. Our data are in accordance with these latter studies in which the rates of expulsion were similar between groups. However, the relatively small sample size in the present study does not allow us to draw firm conclusions.

As discussed above, vaginal delivery is a risk factor for expulsion according to some (but not all) studies. Parity, as well as operator experience, has also been suggested as a risk factor for IUD expulsion [12, 13]. The probable mechanism behind the increased expulsion rate in patients who delivered vaginally is cervical dilation as well as development of the thin lower segment. We assume this is particularly true for partial IUD expulsions. If this were the case, however, patients who had undergone caesarean delivery in active labour would have had a higher risk of IUD expulsion. On the contrary, our results suggest that the impact of vaginal delivery or cervical changes in active labour on IUD expulsion is less evident. Parity, however, increases the risk of IUD expulsion regardless of the mode of delivery. It should also be mentioned that in the current study all the IUDs were placed by experienced physicians, which controls for another potential confounder. Similar conclusions were reached in a study from Mexico, in which the authors found that parity was the only risk factor for expulsion when the IUD was inserted immediately after delivery [12]. On the other hand, a recent study which included only vaginal deliveries found that low parity was associated with a higher expulsion rate. The authors commented that uterine involution was more prominent in primiparous women [13].

### Strengths and weaknesses of the study

The inability to meet the sample size in the power calculation and the drop-out rate of 4.4 % are limitations of the current study. Drop-out of these patients might have been due to complications and the subsequent removal of the IUD. It is also possible that these patients might have chosen to visit a local family planning service, as our clinic is a tertiary referral center to which patients from different provinces are referred. Most of the unelucidated areas in the study are due to the relatively small sample sizes. This invited both type I and type II errors, and yielded large CIs in the logistic regression analysis. Future studies with larger sample sizes are required to assess the risk factors for IUD expulsion in patients with immediate postplacental IUD insertion.

## Conclusions

The findings of the present study confirm previous data that immediate postplacental IUD placement is a viable contraceptive option. Parity but not active labour has been found as a risk factor for IUD expulsion.

## Abbreviations

IUD: Intra uterine device
CI: Confidence interval

## References

[1] Chi I (2003). What we have learned from recent IUD studies: a researcher’s perspective. Contraception.

[2] Fortney JA, Feldblum PJ, Raymond EG (1999). Intrauterine devices. The optimal long-term contraceptive method?. J Reprod Med.

[3] d’Arcangues C (2007). Worldwide use of intrauterine devices for contraception. Contraception.

[4] Bühling KJ, Zite NB, Lotke P, Black K, INTRA Writing Group (2014). Worldwide use of intrauterine contraception: a review. Contraception.

[5] Peterson HB, Xia Z, Hughes JM, Wilcox LS, Tylor LR, Trussell J (1996). The risk of pregnancy after tubal sterilization: findings from the U.S. Collaborative review of sterilization. Am J Obstet Gynecol.

[6] Andersson K, Ryde-Blomqvist E, Lindell K, Odlind V, Milsom I (1998). Perforations with intrauterine devices. Contraception.

[7] Çelen Ş, Sucak A, Yıldız Y, Danışman N (2011). Immediate postplacental insertion of an intrauterine contraceptive device during cesarean section. Contraception.

[8] Zhou SW, Chi IC (1991). Immediate postpartum IUD insertions in a Chinese hospital—a two year follow-up. Int J Gynaecol Obstet.

[9] Lara Ricalde R, Menocal Tobias G, Ramos Pérez C, Velázquez Ramírez N (2006). Random comparative study between intrauterine device Multiload Cu375 and TCu 380a inserted in the postpartum period [in Spanish]. Ginecol Obstet Mex.

[10] Levi E, Cantillo E, Ades V, Banks E, Murthy A (2012). Immediate postplacental IUD insertion at cesarean delivery: a prospective cohort study. Contraception.

[11] Lara R, Sánchez RA, Aznar R (1989). Application of intrauterine device through the incision of the cesarean section [in Spanish]. Ginecol Obstet Mex.

[12] Bonilla Rosales F, Aguilar Zamudio ME, Cázares Montero Mde L, Hernández Ortiz ME, Luna Ruiz MA (2005). Factors for expulsion of intrauterine device Tcu380A applied immediately postpartum and after a delayed period [in Spanish]. Rev Med Inst Mex Seguro Soc.

[13] Jatlaoui TC, Marcus M, Jamieson DJ, Goedken P, Cwiak C (2014). Postplacental intrauterine device insertion at a teaching hospital. Contraception.

[14] Kapp N, Curtis KM (2009). Intrauterine device insertion during the postpartum period: a systematic review. Contraception.

[15] Curry CL, Iverson R, Rindos N, Sonalkar S. Immediate postplacental IUD placement after cesarean and vaginal deliveries at an academic training center [abstract]. Contraception. 2012;86.

